# Can a Humanoid Face be Expressive? A Psychophysiological Investigation

**DOI:** 10.3389/fbioe.2015.00064

**Published:** 2015-05-26

**Authors:** Nicole Lazzeri, Daniele Mazzei, Alberto Greco, Annalisa Rotesi, Antonio Lanatà, Danilo Emilio De Rossi

**Affiliations:** ^1^Research Center “E. Piaggio”, University of Pisa, Pisa, Italy; ^2^Faculty of Psychology, University of Florence, Florence, Italy; ^3^Information Engineering Department, University of Pisa, Pisa, Italy

**Keywords:** facial expressions, emotion perception, humanoid robot, expression recognition, social robots, psychophysiological signals, affective computing

## Abstract

Non-verbal signals expressed through body language play a crucial role in multi-modal human communication during social relations. Indeed, in all cultures, facial expressions are the most universal and direct signs to express innate emotional cues. A human face conveys important information in social interactions and helps us to better understand our social partners and establish empathic links. Latest researches show that humanoid and social robots are becoming increasingly similar to humans, both esthetically and expressively. However, their visual expressiveness is a crucial issue that must be improved to make these robots more realistic and intuitively perceivable by humans as not different from them. This study concerns the capability of a humanoid robot to exhibit emotions through facial expressions. More specifically, emotional signs performed by a humanoid robot have been compared with corresponding human facial expressions in terms of recognition rate and response time. The set of stimuli included standardized human expressions taken from an Ekman-based database and the same facial expressions performed by the robot. Furthermore, participants’ psychophysiological responses have been explored to investigate whether there could be differences induced by interpreting robot or human emotional stimuli. Preliminary results show a trend to better recognize expressions performed by the robot than 2D photos or 3D models. Moreover, no significant differences in the subjects’ psychophysiological state have been found during the discrimination of facial expressions performed by the robot in comparison with the same task performed with 2D photos and 3D models.

## 1 Introduction

Human beings communicate in a rich and sophisticated way through many different channels, e.g., sound, vision, and touch. In human social relationships, visual information plays a crucial role. Human faces convey important information both from static features, such as identity, age, and gender, and from dynamic changes, such as expressions, eye blinking, and muscular micro-movements. The ability to recognize and understand facial expressions of the social partner allows us to establish and manage the empathic links that drive our social relationships.

Charles Darwin was the first to observe that basic expressions, such as anger, disgust, contempt, fear, surprise, sadness, and happiness, are universal and innate (Darwin, 1872). Since the publication of his book “The Expression of the Emotions in Man and Animals” in 1872, a strong debate over the origin, nature, and function of facial expressions has been raised. He hypothesized that some facial and body movements are evolutions of biologically based motor actions (Darwin, 1872). Indeed, human beings are able to recognize faces and read facial expressions almost unconsciously and with little or no effort (LeDoux, 1996).

Due to rapid advances in robotics, computer graphics and artificial intelligence in recent years, social and interactive agents, both physical and virtual, have become common in our daily lives. The rapid growth of robotics science has made possible the development of a new class of empathic machines known as social robots. These innovative robots are used in various fields ranging from entertainment and education to human assistance and health care (Fong et al., 2003; Christensen, 2009). Whether they are physical or virtual, the primary goal of these social agents is to be able to engage people, i.e., to interact and communicate with others by following social behaviors and rules. Therefore, researchers are focusing on the development of more pleasant and user-friendly social agents that are able to reproduce human facial expressions (Pèlachaud, 2000; Breazeal, 2002). Indeed, the ability to express emotions is fundamental for their believability and social capabilities (Bates, 1994; Fong et al., 2003; Siciliano and Khatib, 2008).

Although both physical robots and virtual characters can be emotionally expressive and believable, they differ in a fundamental aspect: the physical embodiment. The debate about whether a physical embodiment is or not an added value for the establishment of social interactions between humans and these social agents is open (Lee et al., 2006) raising the following questions: *Do we really need to build social humanoid robots? Does the physical embodiment increase the believability and emotion conveying capabilities of synthetic agents? Do humanoid robots and virtual characters have the same capability to convey emotions through facial expressions?* These interrogatives are mainly driven by considerations related to the costs of designing and building such agents. Physical robots are usually high-cost products typically used only in academia and research fields. Conversely, virtual characters are widely used in games, storytelling, tutoring, and e-commerce assistance (Gockley et al., 2006).

The esthetic effect of the robot’s physical embodiment has been debated since Masahiro Mori proposed the “Uncanny Valley” hypothesis in the late 1970s (Mori, 1970; Mori et al., 2012). He hypothesized that the acceptance of a humanoid robot increases with its realism but only up to a certain point when the curve suddenly plunges into the uncanny valley. Thus, an observer could lose the sense of affinity and experience an eerie sensation. Nevertheless, Hanson et al. (2005) demonstrated that robots with highly realistic aesthetic cues can induce higher acceptability rates in the involved subjects.

This work is based on the hypothesis that highly anthropomorphic robots with physical embodiment are able to convey expressions more easily and more intuitively than virtual characters. It is known that the form and appearance of a robot can easily raise some sort of social expectations in people (Fong et al., 2003). Therefore, the physical embodiment may help the robot to express its emotions by means of its materialization that is absent in entities shown on a screen. It is also known that humans recognize positive facial expressions faster than the negative ones (Leppänen and Hietanen, 2004) because they do not require the analysis of the entire face. Positive expressions can be usually characterized by a single feature, such as a smiling mouth for happiness (Adolphs, 2002). Our study also investigated whether this phenomenon is valid for facial expressions performed by an affective humanoid.

Physiological signal variations were studied to investigate whether the facial expression recognition task of 2D photos, 3D models, and a physical robot can induce different psychophysiological states in interacting subjects. Indeed, it is already known that autonomic nervous system (ANS) activity variations can be indirectly measured through several sets of physiological signals (Andreassi, 2000). More specifically, heart rate variability (HRV), extracted using an electrocardiogram (ECG), and electrodermal response (EDR) are known to be strongly correlated with subject’s ANS activity and psychophysiological state (Picard, 1997; Zito et al., 2008; Lanatà et al., 2010; Valenza et al., 2013).

Cognitive theories of emotional disorders (Beck, 1976; Bower, 1981) predict that anxiety should be associated with biases favoring the processing of emotional stimuli. Mathews and Mackintosh (1998) suggest that anxiety states are associated with a more pessimistic interpretation of ambiguous stimuli. The effects of heightened, but non-clinical, levels of trait anxiety are also known to be related with the processing of emotional facial expressions. Indeed, in interpretation tasks where ambiguous facial expressions were displayed, high-trait anxious participants were more likely to identify emotional stimuli as frightening than low-trait anxious participants (Richards et al., 2002). In other studies, subjects with high levels of anxiety demonstrated attentional biases toward the identification of both angry (Bradley et al., 1998) and fearful expressions (Fox et al., 2000).

Due to these considerations, an anxiety test was administered to all subjects to avoid biases in recognizing emotional expressiveness. Only subjects with a low or moderate level of anxiety were accepted.

In summary, our study tested the ability of a humanoid robot to perform human perceivable facial expressions in comparison with 2D photos and 3D models of humans and of the robot itself. Moreover, this study also investigated whether the interaction with our humanoid robot induced changes in the subjects’ psychophysiological state. In particular, this research was driven by the following questions:

1. Is a robot able to convey expressions as well as humans 2D photos and 3D models?
2. Are there statistical differences in interpreting facial expressions shown as 2D photos, 3D models, or performed by a physical robot?
3. Studies investigating the recognition of different facial expressions state that human positive emotions are faster and usually simpler to recognize than negative ones. Does the designed protocol verify this phenomenon with 2D photos and 3D models of human expressions?
4. Is the positive/negative expression recognition phenomenon also valid for expressions performed by a humanoid robot?
5. Does the interpretation of humanoid robot expressions induce a different psychophysiological state in comparison with 2D photos and 3D models?

This paper is structured as follows: Section 2 discusses the related work; Section 3 presents materials and methods used in the experiment to create the stimuli, to acquire and analyze physiological signals, and describes the protocol of the experiment and its set-up; Section 4 reports the statistical analysis and related results of the facial expression recognition test and of the subjects’ physiological signals analysis; finally, Sections 5 discusses and summarizes the results of the experiment.

## 2 Related Work

The main question of this study is whether the physical embodiment of a humanoid robot adds value to the establishment of an emotional interaction with humans. Emotional expressiveness has been widely investigated but less attention has been paid to the evaluation of whether there are differences in perceiving expressions between virtual and physical agents.

Bartneck et al. (2004) investigated the influence of the character’s embodiment on how users perceive its emotional expressions. They used iCat, a cartoon-like robot with facial expression capabilities controlled by 13 standard R/C servos, and a computer screen showing a face. The emotion factor consisted of five basic emotional expressions, i.e., anger, fear, happiness, sadness, and surprise, by excluding the disgust expression, which received very low recognition ratings in a pilot test. They found that the embodiment of their character had no significant influence on how people perceived its emotional expression and did not help to express emotions better than the screen character.

Kätsyri and Sams (2008) investigated the comparison between the identification of facial expressions of human faces and a virtual character called Talking Head. The emotional stimuli included the six basic expressions, i.e., anger, disgust, fear, happiness, sadness, and surprise, taken from three different databases: Ekman–Friesen facial affect pictures (Ekman and Friesen, 1976b), Cohn–Kanade database (Kanade et al., 2000), and their own expression database. Each expression was tagged with the correct label and shown to the subjects. The survey investigated the subjects’ agreement with the expression (indicated by the label) using a scale from 1 to 7 (1 = totally disagree, 4 = uncertain, 7 = totally agree). Their preliminary results indicated that the identification of Talking Head stimuli was worse than human stimuli.

In the case of humanoid robots, Becker-Asano and Ishiguro (2011) designed two online surveys to investigate the expressiveness of facial emotions performed by a humanoid robot called Geminoid F. In the first survey, users were asked to label photos of Geminoid F’s face by choosing among angry, fearful, happy, neutral, sad, surprise, or “none of these labels.” They found that people confused the facial expressions of Geminoid F more often than the corresponding human expressions. The second online survey was aimed at investigating whether intercultural differences influence the judgment of an expression. The Asian participants showed lower agreement in labeling the expressions than the American and European participants probably due to general intercultural differences. Indeed, Japanese people tend to focus more on the eyes than the mouth region (Yuki et al., 2007) and the model person’s portrayals showed much more variations around the eyes than Geminoid F, which has limited ability to change its face around the eyes.

In addition to these seminal studies, our experimental work investigated the influence of the physical embodiment on the recognition of facial expressions performed by a very realistic humanoid. The stimuli used in our experiment included human and robot facial expressions shown as 2D photos and 3D models and facial expressions performed by the physical robot in real-time.

The creation of facial expressions that reproduce realistic human facial movements is a very complex process. It involves knowledge of human anatomy, psychology, and engineering and requires many steps, from the analysis of the facial muscles involved in the expression to the mapping of these muscles on the position and intensity of the robot motors. Shayganfar et al. (2012) tried to standardize the mechanism of designing emotional facial expressions using a systematic approach, which is valid for humanoid robots with limited degrees of freedom. They applied this methodology to their humanoid robot and asked people to identify the facial expressions. Their results showed that the predicted data by their methodology qualitatively agreed with the observed data in the static conditions but less in the dynamic conditions due to some aspects of the dynamic expressions not explicitly considered in the design methodology, such as the order of onset of action units and their durations.

Our work used a similar approach to Shayganfar’s study, but it also investigated the effectiveness of robot facial expressions in comparison with human expressions.

Another fundamental aspect concerns the validity of the “uncanny valley” hypothesis, i.e., whether the “uncanny valley” emerges at a certain level of realism.

Tinwell et al. (2011)) investigated the presence of “uncanniness” by comparing animations of human facial expressions with two types of virtual character animations, i.e., fully and partially animated facial expressions. In the partially animated facial expressions, all movements in the top part of the face were disabled. Their results showed that videos of humans were rated as more familiar and human-like than both videos of the virtual character. Moreover, the full animation stimuli attracted higher ratings than those with partial facial animation.

Conversely, Bartneck et al. (2007) showed that there was no strong evidence that supported the uncanny valley hypothesis. Their goals were to attempt to plot Mori’s hypothesized curve and investigate the influence of framing on the user’s perception of the stimuli. They presented three different pictures of six categories, i.e., real and manipulated humans, virtual characters, robots, humanoids, and actroids (humanoid robots with strong visual human-likeness). Their results showed that appealing and acceptance as human-like was not influenced by the category but only by the esthetic appearance. Indeed, highly human-like androids were not uncanny because they were robots, but because of their level of anthropomorphism. On the base of these results, they suggested the existence of an uncanny cliff model rather than an uncanny valley model since even pictures of humans do not reach the level of pictures of toy robots.

Several researchers adopted Mori’s hypothesis as a guideline for the design of the physical appearance of both virtual agents (Fabri et al., 2004; Wages et al., 2004; Hodgkinson, 2009) and physical robots (Cañamero and Fredslund, 2001; DiSalvo et al., 2002; Fong et al., 2003; Minato et al., 2004; Woods et al., 2004). Indeed, this hypothesis could hold true for both virtual agents and physical robots, but the question is still open. This work is not focused on the validation of Mori’s hypothesis but on a comparison between a physical robot and its virtual model, which belongs to this historical debate of the influence of the physical embodiment on the emotion convey capability of affective synthetic agents.

## 3 Materials and Methods

### 3.1 The robot FACE

Facial automaton for conveying emotions (FACE) is an android female face used to study human–robot interactions with a focus on non-verbal communication, developed in collaboration with Hanson Robotics (Hanson, 2006). FACE consists of a passive body equipped with a robotic head made of an artificial skull based on the portrait of a woman covered by a porous elastomer called Frubber™. FACE is animated by 32 servo motors positioned inside the skull and in the upper torso and linked to specific elastomer-fiber anchors with yarns. The mechanical properties of Frubber™ allow it to be stretched by the motion of the servo motors distributing the force in the area close to the anchor point of the skin.

In this study, the attention was focused on recognizing the six basic emotions considered as “universally accepted” by Ekman (1992), e.g., happiness, sadness, anger, fear, disgust, and surprise. Facial expressions of FACE were manually created using an expression editor that is part of Hybrid Engine for Facial Expression Synthesis (HEFES) (Mazzei et al., 2012), a software system devoted to the synthesis and animation of facial expressions of FACE.

**Figure 1:**
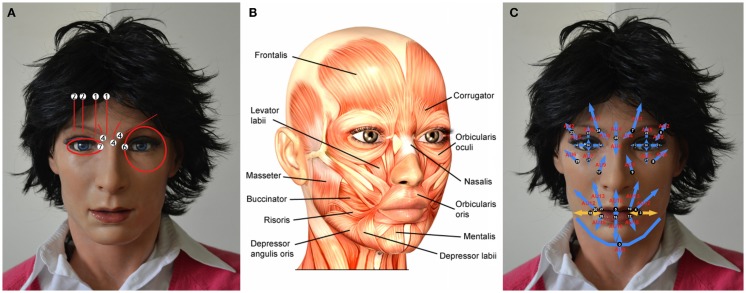
**(A)** AU positions mapped on the robot; **(B)** major facial muscles involved in the facial expressions; and **(C)** servo motor positions corresponding to the Aus.

The Facial Action Coding System (FACS) developed by Ekman and Friesen (1976a) was used to design a set of six standardized basic expressions of FACE. Using the FACS, a facial expression can be decomposed into action units (AUs), which are defined as observable independent movements of the face. The servo motors of FACE are positioned inside the skull and the upper torso similarly to the major facial muscles; therefore, it is possible to find a correspondence between them and the AUs (Figure 1). However, due to a non-perfect mapping between the position and intensity of AUs and the position and strength of the FACE servo motors, FACS-based facial expressions of FACE were less expressive than the ones made following a free hand artistic modeling. Therefore, the FACS-based facial expressions were refined according to Artnatomy^1^, an online visual tool developed at the Fine Arts University of Valencia and based on the atlas of human anatomy. Artnatomy illustrates the underlying anatomical structures of the face and the muscles involved in the different facial expressions. This information was used to identify the servo motors that could take part in each facial movement refining the FACS-based facial expressions of FACE.

### 3.2 Synthetic 2D and 3D stimuli

**Figure 2:**
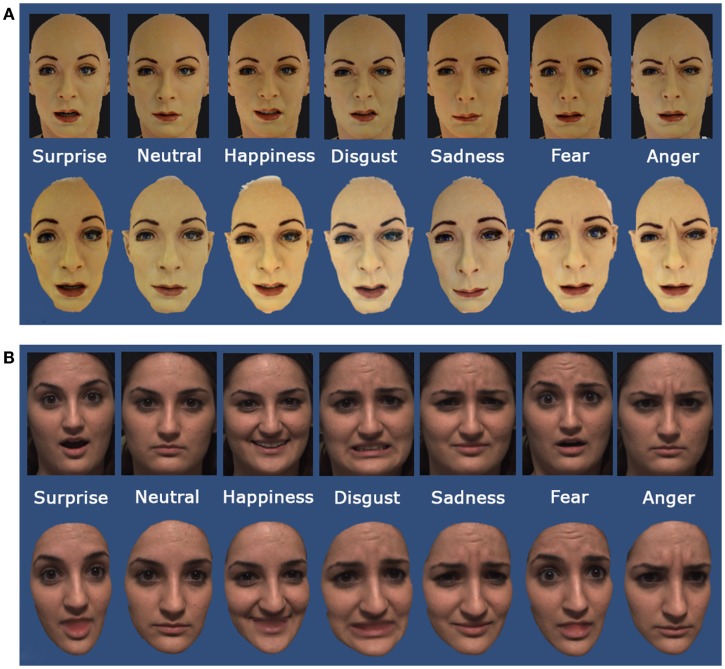
**2D photos and 3D models used in the experiment: (A) FACE expressions and (B) human expressions**.

The stimuli chosen for the experiment were 2D photos and 3D models of the robot FACE and 2D photos and 3D models of a human being (Figure 2). A photography of each basic expression was used to create the set of 7 images for FACE. The set of 7 3D models of FACE was created using the Autodesk^®^ 123D^®^ Catch program, which generates 3D models by taking a set of pictures acquired from various angles as inputs. The set of FACE pictures included one hundred photos taken around the robot, covering approximately 180°.

The set of human 2D photos and 3D models was taken by selecting a single subject from the Bosphorus Database (Savran et al., 2008). The Bosphorus Database is a 2D/3D collection of FACS-based facial expressions acquired using a structured-light based 3D system (Savran et al., 2012). The subject selected from the Bosphorus Database was a female with Caucasian traits (item bs103). The Unity 3D software (Unity Technologies, 2013) was used as the front-end animation tool to show both 2D photos and 3D models.

As previously mentioned in Section 1, FACE servo motors are not perfectly mapped on AUs. As shown in Table 1, a similar problem is also present in the Bosphorus database, which used an adapted mapping. The AU configurations taken as reference point for human expressions are reported in Matsumoto and Ekman (2008). Table 1 shows a comparison between the Ekman AUs configuration, the adapted AUs configuration for FACE, and the adapted AUs configuration used in the Bosphorus database.

**Table 1:** **Comparison between the action unit configurations of the expressions: the Ekman AUs configuration (first column), the adapted AUs configuration for FACE (second column), and the adapted AUs configuration used in the Bosphorus database (third column)**.

	Action units		
	FACS based on humans	FACE	Bosphorus DB
Anger	4 + 5 + 7 + 22 + 23 + 24	4B + 7A + 16E + 25C	4C + 38A
Disgust	9 + 10 + (25/26)	4B + 7B + 9D + 10D + 16E + 25C	1C + 4B + 7B + L10A + R11C + 20C + 25C
Fear	1 + 2 + 4 + 5 + 20 + 26	1C + 2C + 4B + 5D + 20B + 26B	1C + R2C + 5D + 25C + 26C + 38C
Happiness	6 + 12	6E + 7B + 12E + 25D	7B + 10C + 12C + 25D
Sadness	1 + (4) + 15 + (17)	1D + 2A + 4E + 7B + 15E	1C + 4B + 7C + 11C
Surprise	1 + 2 + 5 + 25/26	1E + 2E + 5D + 12C + 25D + 27D	1B + 2B + 5B + 25C + 27C

Due to technical problems with the servo motor corresponding to the buccinator muscle (motor *no*. 4), FACE was partially unable to raise the left part of the smile obtaining an ambiguous happiness expression.

### 3.3 Experimental set-up

Participants were seated comfortably at a desk within a room, approximately 0.5 m from either from a TV screen (size: 32″, frame rate: 100 Hz, resolution: 1920 × 1080) or the robot. The experimental set-up included three laptops, one for controlling the robot FACE, one for controlling the animation on the TV screen, and the last one for acquiring the subjects’ physiological signals.

### 3.4 Subjects’ recruitment

Fifteen subjects (10 males, 5 females) aged 19–31 years (mean age 24.1 ± 3.4) were recruited for the experiment. All subjects, except for one, studied scientific disciplines at the University of Pisa (IT). All participants were native Italian speakers and had either normal or corrected vision. All subjects gave written informed consent for participation.

### 3.5 Experimental protocol

Anxiety can bring a bias in emotion recognition test, as reported in several studies (Rossignol et al., 2005; Cooper et al., 2008), where they assumed that anxious subjects have an attention bias for fearful faces. In the study of Richards et al. (2002), ambiguous emotional facial expressions were identified as fearful from high-trait anxious participants.

To study the influence of anxiety in our emotion recognition tasks, all subjects completed both the trait and state sections of the Spielberger State-Trait Anxiety Inventory (STAI) (Spielberger et al., 1983). It differentiates between the temporary condition of “state anxiety” and the more general and long-standing quality of “trait anxiety.” The trait subtest was administered to the subjects some days before the experiments while the state subtest was administered immediately before the beginning of the experiment. The state section was considered a control test.

Subjects with scores above the 75th percentile or below the 25th percentile on the trait component of the STAI are considered high or low anxiety as reported in the previous study by Cooper et al. (2008). All subjects had a trait anxiety level under the 75th percentile and over the 25th percentile (males: 37 ± 10.68; females: 43.4 ± 12.28); therefore, no subjects were considered high-trait or low-trait anxious.

Before the beginning of the protocol, each subject signed the informed consent form and was instructed about the rules and times of the experiment. During the entire experiment, the subjects’ physiological signals were continuously monitored.

The protocol was structured as a stepwise stimulation where the realism of the stimulus gradually increases. In particular, the presentation of the stimuli was organized according to the following four phases:

- Rest phase: 3 min of rest to acquire the physiological signal baseline.
- First phase: each subject had to recognize 14 2D photos of facial expressions: 7 photos of humans from the Bosphorus database and 7 photos of FACE, in random order (different for each subject).
- Second phase: each subject had to recognize 14 3D models of facial expressions: 7 3D models of humans from the Bosphorus database and 7 3D models of FACE, in random order (different for each subject).
- Third phase: each subject had to recognize six basic expressions performed by the robot FACE in random order (different for each subject).

The set of possible answers included the seven basic expressions available in the database and reproduced by the robot (indicated by * in Table 2) plus eight similar labels and “I do not know.” The set of possible answers was extended to avoid a strictly forced-choice method and allow subjects to choose the label that best matched the expression in their opinion, as suggested in Russell’s review of the cross-cultural studies about the recognition of emotion from facial expressions (Russell, 1994).

**Table 2:** The set of possible answers for all phases which includes the 7 basic expressions available in the database and reproduced by the robot (indicated by * symbol).

*Italian*	English
***Orgoglio***	Pride
Felicità*	Happiness*
***Imbarazzo***	Embarrassment
Neutra*	Neutral*
Sorpresa*	Surprise*
Disgusto*	Disgust*
***Dolore***	Pain
***Compassione***	Pity
***Disprezzo***	Contempt
Tristezza*	Sadness*
***Interesse***	Interest
***Vergogna***	Shame
Paura*	Fear*
***Eccitamento***	Excitement
Rabbia*	Anger*
***Non lo so***	I do not know

*Italian answers are in bold style, English translation is in normal style*.

In each phase, the subject had a total of 30 s to recognize the expression and give the answer, i.e., at most 10 s to observe the expression plus at most 20 s to answer. In all three phases, after 30 s, the next stimulus appeared. If no answer is given, the software assigned a null label that was considered as an incorrect answer.

In the first and second phase, after the first 10 s or if the subject pressed “Enter” a black screen appeared and the subject had to choose one of the possible answers from the questionnaire. The response time was recorded on the “Enter” key pressing.

In the third phase, after the first 10 s or before the subject answered by selecting a label of the questionnaire shown on the screen, the robot performed the neutral expression. In this case, the subject evaluated six instead of seven different facial expressions because the neutral expression was used as “black screen.” The subject had to select an option directly on the screen through a software tool running on a laptop. The response time was recorded by the mouse click.

The maximum duration of the experiment was 20 min.

To avoid the subjects’ loss of interest and attention due to the length of the experiment and the repetitiveness of the tasks, the protocol included only one initial rest phase and physiological signal variations of all phases were normalized to the initial rest phase.

### 3.6 Physiological signals acquisition and analysis

The autonomic nervous system variations induced by emotional elicitation was investigated through the processing of both electrocardiogram (ECG) and skin conductance (SC) tests. These biosignals are used to infer if the interpretation of robot expressions induced different psychophysiological states in the subjects in comparison with the interpretation of human 2D photos and 3D models of facial expressions. In this work, the psychophysiological measures were used as indicators of the general autonomic nervous system activity (Rani et al., 2002; Bethel et al., 2007; Mauri et al., 2010) and not as indicators of the elicited arousal or affective level.

The two biosignals, i.e., ECG and SC, were continuously acquired using BIOPAC MP150 instrumentation, which was equipped with body-worn sensors. Specifically, the ECG100C analog front-end was used for the ECG acquisition and the GSR100C analog front-end was used for the skin conductance measure.

To acquire the ECG, three pregelled Ag/AgCl electrodes were placed following the Einthoven triangle configuration. This signal was used only to extract the heart rate variability (HRV) because it is well known that the HRV carries information about how physiological factors, more specifically the sympathetic/parasympathetic balance and the normal heart-beat rhythm (Rajendra Acharya et al., 2006). In particular, the HRV estimation was extracted from the QRS complexes using the Pan–Tompkins algorithm (Fowles et al., 1981). The *R*–*R* time intervals (tachogram) were calculated as the time difference between consecutive *R*-peaks. Later, the HRV was computed using a cubic spline interpolation and the problem of the resulting non-uniform sampling was overcome by a resampling at 4 Hz (Valenza et al., 2012).

The SC signal was acquired through two Ag/AgCl electrodes placed at the fingertip of the middle and index fingers of the non-dominant hand. In the scientific literature, SC variations are known to be psychologically induced changes in sweat gland activity due to external affective stimuli (Winton et al., 1984). In fact, SC is widely accepted and used as an indirect autonomic nervous system (ANS) marker because the sympathetic nerve activity (SNA) directly controls the sweat gland activity (Boucsein, 1992). The signal processing methodology used for treating SC was mainly based on the decomposition of SC into a phasic and tonic component (Greco et al., 2012, 2014). The tonic component, also known as the skin conductance level (SCL), is the baseline signal and is different from person to person. The SCL depends on both the patient physiological state and autonomic regulation. By contrast, the phasic component, also known as the skin conductance response (SCR), is superimposed on the SCL and is directly related to the external stimuli. Here, following the experimental set-up and the timeline of the stimulation protocol, only the SCR component was used.

The HRV and SCR signals were divided into sections according to the experimental protocol and several features were extracted. Specifically, the HRV features were extracted in both the time and frequency domains. The time domain features included statistical parameters and morphological indexes. Because the changes in the spectral power are known to be correlated with the ANS modulation (Camm et al., 1996), the frequency domain features included the calculation of three spectral powers for the following bands: very low frequency (VLF), low frequency (LF), and high frequency (HF) components. The set of HRV features is reported in Table 3. After the feature extraction, a statistical analysis was performed on the obtained dataset.

**Table 3:** **HRV and SCR features extracted from the subjects’ physiological signals**.

HRV features				SCR features	
Time domain		Frequency domain	
Mean RR	Mean inter-beat interval (in ms)	VLF	Very low frequency (in Hz and in ms^2^)	nSCR	Number of SCR in the windows response
STD RR	Standard inter-beat interval (in ms)	LF	Low frequency (in Hz and in ms^2^)	MAX-phasic	Maximum value of the phasic component
RMSSD	Root mean square of the successive differences (in ms)	HF	High frequency (in Hz and in ms^2^)	Latency	Time interval between the stimulus and the SCR peak
NN50	Number of pairs of successive beat-to-beat intervals (NNs) that differ by more than 50 ms (count)	LF/HF ratio	Ratio between the power of LF and HF bands indicating the balance between sympathetic and parasympathetic systems	AUC-phasic	Area under the phasic curve over time
pNN50	Proportion of NN50 divided by total number of NNs (in %)	LF	Ratio between absolute value of the LF and difference between total power and VLF (in n.u., i.e., normalized unit)	Mean-phasic	Mean value of the phasic component
HRV tri-index	Total number of all NN intervals divided by the height of the histogram of all NN intervals	HF	Ratio between absolute value of the HF and difference between total power and VLF (in n.u., i.e., normalized unit)	STD-phasic	SD of the phasic component

## 4 Results

### 4.1 Data analysis

The set of expressions considered in the analysis included anger, disgust, fear, sadness, and surprise. As mentioned in Section 3.2, the happiness expression of the robot was ambiguous due to technical problems with the servo motors. For this reason, the happiness expression was excluded from all datasets in the data analysis.

The facial expression recognition rates were analyzed using the Cohen’s kappa (Cohen, 1960), a statistical measure of inter-rater reliability used to examine the agreement among observers on categorizing a clustered variable. The Cohen’s kappa ranges from −1.0 to 1.0, where large numbers mean better reliability, values near zero suggest that agreement is attributable to chance, and values <0 signify that agreement is even less than that which could be attributed to chance. According to Landis and Koch (1977), with a significance level of 0.05, kappa can be classified according to the following: *k* ≤ 0.00 less than chance agreement, 0.01 < *k* < 0.20 slight agreement, 0.21 < *k* < 0.40 fair agreement, 0.41 < *k* < 0.60 moderate agreement, 0.61 < *k* < 0.80 substantial agreement, and 0.81 < *k* ≤ 1 almost perfect agreement.

The Kolmogorov–Smirnov test (Smirnov, 1948) and the analysis of variance were applied to the datasets of the response times and physiological signals. When normality condition and homogeneity of variance within each of the populations were confirmed, the ANOVA-1way parametric test with a *post hoc* Bonferroni test (Scheffé, 1999) was used to examine the category differences. Otherwise, the Kruskall–Wallis test (Kruskal and Wallis, 1952) was preferred due to the non-parametric nature of the gathered data, i.e., the variables were not normally distributed or the variance within each of the populations was not equal. The statistical inference was carried out using the OriginLab software (OriginLab, 2012).

Data analysis was carried out aiming at discussing the research interrogatives at the base of this work.

#### 4.1.1 Are Facial Expressions of the Robot Perceived as Well as the Expressions of the Humans? Yes

The subjects’ answers for the human 2D photos, human 3D models, and FACE robot expressions are reported as confusion matrix (i.e., a specific table, which contains information about the presented models (on the columns) against the selected labels (on the rows) in Table 4. For all three categories, the best recognition rate was achieved for the surprise expression. Human anger was not so well understood, i.e., it was classified as “I do not know” (33.33% in the human 2D photos and 20% in the human 3D models). The human disgust was often labeled as “pain” (26.67% in the human 2D photos and 33.33% in the human 3D models) or as “contempt” (20% in the robot expressions). Fear was confused with “surprise” both in the human 2D photos (60%) and in the human 3D models (53.33%). Moreover, the expression intended to convey sadness was labeled as “pity” (33.33% in the human 2D, 53.33% in the robot expressions) or “pain” and “embarrassment” (20% in the human 3D models). The Cohen’s kappa of the three categories showed a homogeneous expression evaluation: *K_Hum_*_2_*_D_* = 0.536 (*p* < 0.001) 95% CI (0.338, 0.733), *K_Hum_*_3_*_D_* = 0.616 (*p* < 0.001) 95% CI (0.440, 0.791), and *K_Robot_* = 0.648 (*p* < 0.001) 95% CI (0.491, 0.805).

**Table 4:** **Confusion matrix (*N* = 15) of the recognition rates (in percentage) of the seven (for humans) and six (for the robot) facial expressions with presented models (columns) against selected labels (rows)**.

Confusion matrix (*N* = 15)																	
	Human 2D photos						Human 3D models						Physical robot				
	A	D	F	N	Sa	Su	A	D	F	N	Sa	Su	A	D	F	Sa	Su
Anger	13.3	6.7	0.0	0.0	0.0	0.0	**33.3**	0.0	0.0	0.0	0.0	0.0	**33.3**	20.0	0.0	0.0	0.0
Disgust	0.0	**33.3**	0.0	0.0	13.3	0.0	0.0	20.0	6.7	0.0	0.0	0.0	13.3	**53.3**	13.3	0.0	6.7
Fear	0.0	0.0	20.0	0.0	0.0	0.0	6.7	20.0	26.7	0.0	0.0	0.0	6.7	0.0	**53.3**	0.0	20.0
Neutral	0.0	0.0	0.0	**86.7**	0.0	0.0	0.0	0.0	0.0	**86.7**	0.0	0.0	/	/	/	/	/
Sadness	0.0	13.3	0.0	6.7	20.0	0.0	0.0	0.0	0.0	6.7	**40.0**	0.0	0.0	0.0	0.0	33.3	0.0
Surprise	0.0	0.0	**60.0**	0.0	0.0	**93.3**	0.0	0.0	**53.3**	0.0	0.0	**93.3**	0.0	0.0	13.3	0.0	**73.3**
Pride	6.7	0.0	0.0	0.0	0.0	0.0	13.3	0.0	0.0	0.0	0.0	0.0	6.7	0.0	0.0	0.0	0.0
Embarrass.	0.0	6.7	13.3	0.0	0.0	6.7	0.0	13.3	6.7	0.0	20.0	0.0	0.0	0.0	13.3	0.0	0.0
Pain	6.7	26.7	0.0	0.0	13.3	0.0	0.0	**33.3**	6.7	0.0	20.0	0.0	13.3	0.0	0.0	13.3	0.0
Pity	0.0	6.7	0.0	0.0	**33.3**	0.0	6.7	0.0	0.0	0.0	13.3	0.0	0.0	6.7	0.0	**53.3**	0.0
Contempt	20.0	0.0	0.0	6.7	6.7	0.0	6.7	0.0	0.0	6.7	0.0	0.0	20.0	20.0	0.0	0.0	0.0
Interest	20.0	0.0	0.0	0.0	0.0	0.0	13.3	0.0	0.0	0.0	0.0	0.0	0.0	0.0	0.0	0.0	0.0
Shame	0.0	6.7	0.0	0.0	0.0	0.0	0.0	6.7	0.0	0.0	6.7	0.0	0.0	0.0	0.0	0.0	0.0
Excitement	0.0	0.0	0.0	0.0	0.0	0.0	0.0	0.0	0.0	0.0	0.0	6.7	0.0	0.0	0.0	0.0	0.0
Idonotknow	**33.3**	0.0	6.7	0.0	13.3	0.0	20.0	6.7	0.0	0.0	0.0	0.0	0.0	0.0	6.7	0.0	0.0
Noanswer	0.0	0.0	0.0	0.0	0.0	0.0	0.0	0.0	0.0	0.0	0.0	0.0	6.7	0.0	0.0	0.0	0.0

*The highest values are set in bold*.

*The column labels are A, anger; D, disgust; F, fear; N, neutral; Sa, sadness; Su, surprise*.

**Figure 3:**
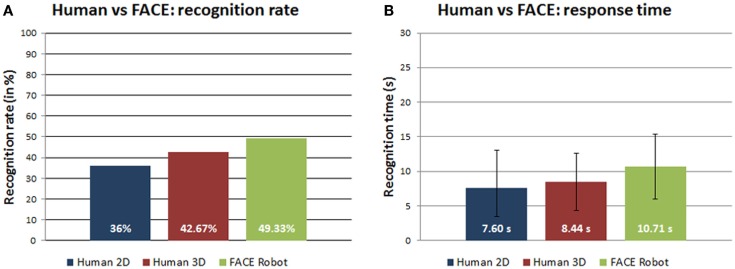
**(A)** Recognition rates (in percentage) and **(B)** response time (in seconds) of human 2D photos, human 3D models, and robot FACE expressions.

Figure 3A shows a trend of increasing recognition rate for stimuli that gradually become more realistic, i.e., from human photographs to human 3D models up to the physical robot. This supports our hypothesis about the importance of the physical embodiment in conveying expressions.

Table 5 shows the means and the SDs of the response time for each expression in the three categories: human 2D photos, human 3D models, and FACE robot. The ANOVA-1way parametric test did not find significant differences between the three categories [*F*(2,89) = 1.7584, *p* = 0.17825, α = 0.05] (Figure 3B).

**Table 5:** **Means and SDs of the response time (in seconds) of 15 subjects in recognizing the facial expressions of human 2D photos, human 3D models, and the robot FACE**.

	Response time (s)					
	Human 2D		Human 3D		Robot	
	Mean	SD	Mean	SD	Mean	SD
Anger	4.09	0.60	8.42	5.71	8.53	4.43
Disgust	7.42	4.26	10.79	3.30	10.55	5.78
Fear	9.71	6.90	11.01	1.61	9.68	3.87
Sadness	15.40	8.76	9.78	5.45	16.02	0.96
Surprise	6.81	5.48	8.39	3.83	9.31	4.20

#### 4.1.2 Is there a Valid and Useful Reason to Create and Develop a Realistic Humanoid Robot Instead of Using Photographs or 3D Model? Yes

Our study is based on the hypothesis that the physical embodiment of the FACE robot is an added value that could help people to better understand and interpret its emotional state.

Table 6 shows the confusion matrix of the subjects’ answers for the robot expressions shown as 2D photos, 3D models, and physical robot. As in the previous case, for all three categories, the best recognition rate was achieved for the surprise expression. The anger was often confused with “disgust” (46.67% in the 2D photos) or “contempt” (40% in the 3D models), while the disgust was often labeled as “contempt” (46.67% in the 2D photos and in the 3D models). The subjects’ judgments showed less confusion for the fear, neutral, and surprise than the other expressions. Except for the sadness (labeled as “pity” for 53.33% in the expressions performed by the physical robot), the expressions performed by the robot were less confused than those shown in 2D photos or 3D models. The level of agreement between the subjects was comparable for the three categories: *K_FACE_*_2_*_D_* = 0.514 (*p* < 0.001) 95% CI (0.333, 0.693), *K_FACE_*_3_*_D_* = 0.638 (*p* < 0.001) 95% CI (0.456, 0.819), and *K_Robot_* = 0.648 (*p* < 0.001) 95% CI (0.491, 0.805).

**Table 6:** **Confusion matrix (*N* = 15) of the recognition rates (in percentage) of the robot facial expressions with presented models (columns) against selected labels (rows)**.

Confusion matrix (*N* =15)																	
	FACE 2D photos						FACE 3D models						Physical robot				
	A	D	F	N	Sa	Su	A	D	F	N	Sa	Su	A	D	F	Sa	Su
Anger	13.3	6.7	6.7	0.0	0.0	0.0	13.3	20.0	0.0	0.0	0.0	0.0	**33.3**	20.0	0.0	0.0	0.0
Disgust	**46.7**	33.3	13.3	0.0	0.0	13.3	26.7	33.3	0.0	0.0	0.0	0.0	13.3	**53.3**	13.3	0.0	6.7
Fear	6.7	0.0	**33.3**	0.0	0.0	0.0	0.0	0.0	**33.3**	0.0	0.0	0.0	6.7	0.0	**53.3**	0.0	20.0
Neutral	0.0	6.7	0.0	**40.0**	0.0	0.0	0.0	0.0	0.0	**46.7**	0.0	0.0	/	/	/	/	/
Sadness	0.0	0.0	0.0	0.0	**40.0**	6.7	0.0	0.0	0.0	0.0	**33.3**	0.0	0.0	0.0	0.0	33.3	0.0
Surprise	6.7	0.0	6.7	6.7	0.0	**53.3**	6.7	0.0	20.0	0.0	0.0	**73.3**	0.0	0.0	13.3	0.0	**73.3**
Pride	0.0	0.0	0.0	20.0	0.0	0.0	0.0	0.0	0.0	13.3	0.0	0.0	6.7	0.0	0.0	0.0	0.0
Embarrass.	0.0	0.0	13.3	0.0	6.7	6.7	0.0	0.0	13.3	0.0	0.0	6.7	0.0	0.0	13.3	0.0	0.0
Pain	0.0	0.0	0.0	0.0	0.0	0.0	0.0	0.0	0.0	0.0	13.3	0.0	13.3	0.0	0.0	13.3	0.0
Pity	0.0	0.0	6.7	0.0	**40.0**	6.7	0.0	0.0	26.7	0.0	13.3	0.0	0.0	6.7	0.0	**53.3**	0.0
Contempt	13.3	**46.7**	6.7	6.7	6.7	6.7	**40.0**	**46.7**	0.0	6.7	0.0	0.0	20.0	20.0	0.0	0.0	0.0
Interest	0.0	0.0	0.0	20.0	6.7	0.0	13.3	0.0	0.0	26.7	6.7	6.7	0.0	0.0	0.0	0.0	0.0
Shame	0.0	0.0	6.7	0.0	0.0	0.0	0.0	0.0	0.0	0.0	0.0	0.0	0.0	0.0	0.0	0.0	0.0
Excitement	0.0	0.0	0.0	0.0	0.0	0.0	0.0	0.0	0.0	0.0	0.0	0.0	0.0	0.0	0.0	0.0	0.0
I do not know	6.7	6.7	6.7	6.7	0.0	6.7	0.0	0.0	6.7	6.7	26.7	13.3	0.0	0.0	6.7	0.0	0.0
No answer	6.7	0.0	0.0	0.0	0.0	0.0	0.0	0.0	0.0	0.0	0.0	0.0	6.7	0.0	0.0	0.0	0.0

*The highest values are set in bold*.

*The column labels are A, anger; D, disgust; F, fear; N, neutral; Sa, sadness; Su, surprise*.

**Figure 4:**
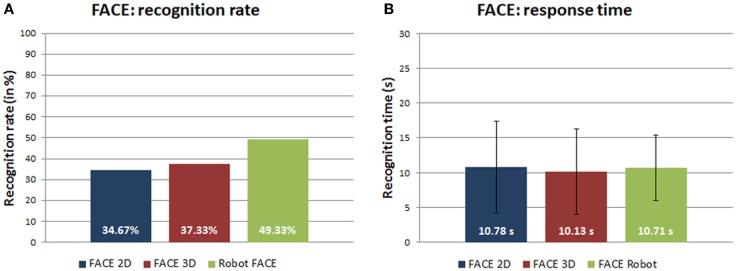
**(A)** Recognition rates (in percentage) and **(B)** response time (in seconds) of robot 2D photos, robot 3D models, and robot FACE expressions.

The comparison among robot stimuli where the realism gradually increases, i.e., from photographs to 3D models up to the physical robot, shows a positive trend of the recognition rate (Figure 4A). This suggests that the embodiment allows the physical robot to better convey the expressions in comparison with its 2D photos and 3D models.

Table 7 shows the response time means and SDs for each expression in the different categories of FACE: 2D photos, 3D models, and the physical robot. The ANOVA-1way parametric test could not distinguish between the three distributions [*F*(2,87) = 0.20964, *p* = 0.81128, α = 0.05] (Figure 4B).

**Table 7:** **Means and SDs of the response time (in seconds) of 15 subjects in recognizing the facial expressions of robot 2D photos, robot 3D models, and the physical robot**.

	Response time (s)					
	FACE 2D		FACE 3D		Robot	
	Mean	SD	Mean	SD	Mean	SD
Anger	16.49	11.20	12.60	9.09	8.53	4.43
Disgust	9.80	9.31	7.46	2.98	10.55	5.78
Fear	7.75	5.90	10.25	8.01	9.68	3.87
Sadness	9.19	5.71	9.12	6.22	16.02	0.96
Surprise	12.86	3.10	10.28	5.83	9.31	4.20

#### 4.1.3 The Literature Studies Investigating the Recognition of Different Facial Expressions State that Positive Emotions are Faster and may be Considered Simpler to Recognize than Negative Facial Expressions. Does our Experiment Confirm this Result? Yes, Partially

In order to investigate if our protocol was able to confirm the phenomenon of positive/negative expression discrimination, the recognition rate and speed of human expressions were analyzed. As already mentioned, the happiness expression of the robot had a technical problem that compromised its interpretation. For this reason, in order to uniform the dataset, the happiness has been removed in this analysis. Therefore, the category of the positive expressions includes only surprise.

**Figure 5:**
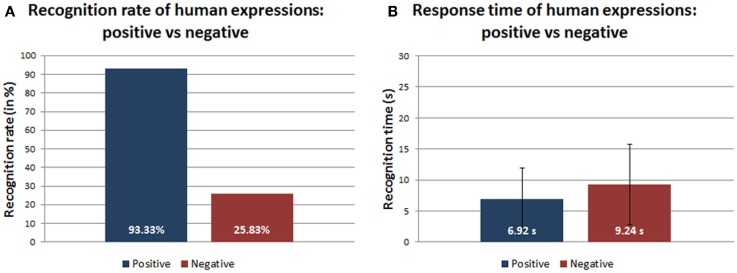
**(A)** Recognition rates (in percentage) and **(B)** response time (in seconds) of positive/negative human expressions.

Figure 5A shows a tendency to better recognize human positive expressions in comparison with human negative expressions. The ANOVA-1way did not show a significant difference between the answer times for the human positive and negative expressions [*F*(1,54) = 2.17483, *p* = 0.14609, α = 0.05] (Figure 5B).

#### 4.1.4 Is the Positive/Negative Recognition Phenomenon Replicated by the Robot? Yes, Partially

**Figure 6:**
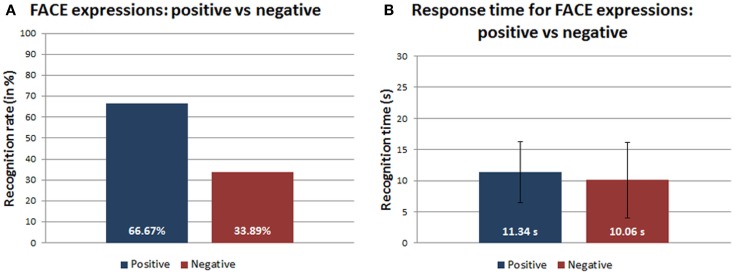
**(A)** Recognition rates (in percentage) and **(B)** response time (in seconds) of positive/negative robot expressions.

Even for the robot stimuli, the discrimination between positive and negative expressions showed a trend to better recognize the positive expressions (Figure 6A). The ANOVA-1way did not show statistically significant differences for the response time between the positive and the negative expressions performed by FACE [*F*(1,88) = 0.18989, *p* = 0.66408, α = 0.05] (Figure 6B).

**Figure 7:**
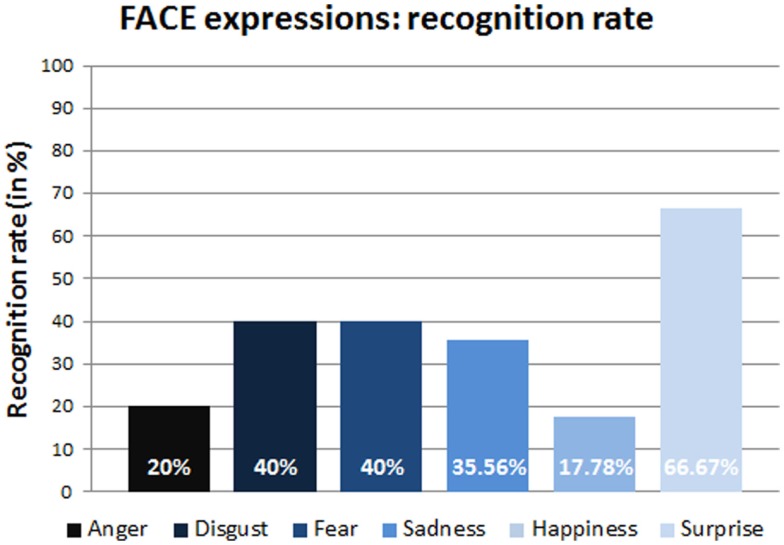
**Recognition rates (in percentage) of the FACE expressions**.

As mentioned at the beginning of the Section 1, the happiness expression performed by the robot resulted ambiguous and difficult to recognize. Figure 7 highlights this fact showing that the happiness is the least recognized expression with a recognition rate of only 17.78%. Instead, the surprise expression achieved the best recognition rate in comparison with each negative expression with a difference of at least 25% (Figure 7).

#### 4.1.5 Does the Interpretation of Humanoid Robot Expressions Induce a Different Psychophysiological State in Comparison with 2D Photos and 3D Models? No

The features extracted from the HRV and SCR physiological signals were statistically analyzed to identify the differences in the subjects’ psychophysiological state induced by the protocol phases.

To assess if the facial expression interpretation task based on 2D photos, 3D models, and the physical robot induces statistically discernible psychophysiological states, the extracted features were studied using the Kruskal–Wallis test due to the non-Gaussianity of samples.

Two different analyses were performed:

- an intra-subject analysis by comparing the results of each subject between the 2D photos, 3D models, and FACE robot stimuli;
- an inter-subject analysis carried out for each expression by grouping the results of all subjects and comparing the responses of the three types of stimuli.

None of the statistical analysis yielded significant differences neither in the intra-subject case nor in the inter-subject case for all features (*p* > 0.05). More specifically, in the intra-subject analysis taking in account each single subject and feature extracted from both the HRV and the SCR analysis, the three groups of data do not show any significant difference. The same results were achieved in the inter-subject analysis, where none feature was able to find any significant difference among the three groups.

**Figure 8:**
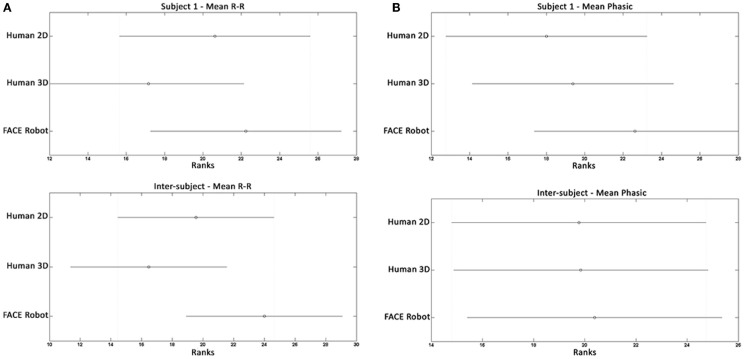
**Statistical analysis of two features extracted from HRV and SCR during the interpretation task based on human 2D photos, human 3D models, and the physical robot**. **(A)** HRV results. Example of an intra-subject (subject 1) and an inter-subject statistical analysis result. The mean RR feature represents the mean value of the RR distance (ms). **(B)** SCR results. Example of an intra-subject (subject 1) and an inter-subject statistical analysis result. The mean-phasic feature represents the mean value of the SCR signal (uSiemens).

An example of statistical result of the interpretation task based on human 2D photos, human 3D models, and the physical robot is shown in Figure 8: the Kruskal–Wallis test on two features extracted from HRV and from SCR highlights the inability to distinguish the three groups both in the intra-subject analysis (Figure 8, top charts) and the inter-subject analysis (Figure 8, bottom charts).

## 5 Discussion

Our study aimed at investigating if: (1) the recognition rates of facial expressions performed by FACE were similar to the ones achieved with humans stimuli; (2) there were differences in recognizing facial expressions performed by FACE shown as 2D photos, 3D models, or by the physical robot itself; and (3) the literature claims that positive emotions are easier and quicker to be recognized than negative ones, is this phenomenon replicated by our protocol with human facial expressions? What happens in the case of robot facial expressions? In addition, the analysis of subjects’ physiological signals has been addressed for discussing how these points can influence the subjects’ psychophysiological state.

The final dataset used in the analysis did not include the happiness expression due to an abnormal functioning of the servo motor related to the mouth of the robot that made the expression ambiguous. The inclusion of the happiness expression could distort the final results as shown in Figure 7.

In regard to the first question, the preliminary results showed a general trend to better recognize expressions performed by the physical robot in comparison with the human stimuli. This supports our hypothesis that the robot is able to convey expressions at least as well as human 2D photos and 3D models. More in detail, anger, disgust, and fear were definitely recognized better when performed by the physical robot. Robot surprise was mainly confused with fear, which can be explained by the fact that these two expressions are characterized by the common motion of Upper Lid Raiser (AU5) and Jaw Drop (AU26) followed by the later activation of Eyebrow Raiser (AU1-2) and the artificial nature of the robot could make this transition less natural than in a human face (Jack et al., 2014). Instead, in all three categories, sadness was often confused with other negative expressions belonging to the same emotional area and with a similar emotional meaning, i.e., pity or pain, and this can be due to the wide range of possible answers, which was extended to allow subjects to choose the label that best matched the expression in their opinion as suggested by Russell (1994). Moreover, the analysis of the response time of human and robot facial expressions did not show significant differences.

Concerning the second question, the recognition rate shows an increasing tendency to better discriminate expressions performed by the robot in comparison with its 2D photos and its 3D models. Even in this case, all expressions were definitely recognized better when performed by the physical robot with the exception of sadness that was often confused with other negative expressions belonging to the same emotional area and with a similar emotional meaning, i.e., pity or pain, in all three categories. As in the previous case, the reason could be the wide forced-choice method adopted and the subjects’ cultural background (Russell, 1994). We can consequently state that the physical embodiment of the robot is an added value for the emotion conveying capability of social humanoids. On the other side, the subjects’ performance in terms of response time in the three categories did not show significant differences.

Regarding the third and fourth question, our results confirm that there is a trend to better recognize positive facial expressions than the negative ones. Indeed, in case of human 2D photos and 3D models, negative expressions were often confused with other expressions of the same category, e.g., disgust with pain or contempt, sadness with pity or pain, except for fear, which was often labeled as surprise. Instead, the statistical analysis of the response time for the human expression recognition did not show significant differences for the two categories, i.e., human 2D photos and 3D models. These results confirm that our protocol partially reproduced the positive/negative expression phenomenon. Even in the case of the physical robot, the result is partially confirmed: the best recognition rate was generally achieved for positive expressions, while negative expressions, such as anger and disgust, were often confused with other negative expressions. Looking at the robot expressions in detail, the surprise was definitely the best recognized emotion whereas, as already mentioned, the happiness was excluded from the dataset due to its ambiguous interpretation as highlighted in Figure 7. Among the negative expressions, the least discriminated expression was anger, which was mainly confused with contempt and with disgust or pain. The reason for the confusion about the anger can be mainly due to the wide range of possible answers of the extended forced-choice method and to the fact that all these emotions belong to the same negative emotional area. Even in this case, the statistical analysis of the response time did not show significant differences between positive and negative expressions of the robot.

In the matter of the last question, the statistical analysis of the subjects’ psychophysiological measures did not show any significant differences neither in the intra-subject case nor in the inter-subject case for the all extracted features. In particular, the intra-subject and inter-subject statistical analyses of the physiological signals could not distinguish between the human 2D photos, human 3D models, and FACE robot facial expressions during the interpretation tasks. These results confirm that the robot is able to perform facial expressions that are perceived as well as human expressions shown as 2D photos or 3D models without altering the subjects’ psychophysiological state. Even in the interpretation of the robot facial expressions shown as 2D photos, 3D models, or performed by the robot itself, the intra-subject and inter-subject analyses of the physiological signals did not show significant differences between the three different tasks. Therefore, we can conclude that interpreting the expressions performed by a physical robot do not induce psychophysiological states that can be classified as different than the ones induced by interpreting facial expressions shown as 2D photos or 3D models.

In conclusion, these results support the contention that the embodiment of physical social humanoids improves the recognition and discrimination rate of emotions in comparison with 2D picture and 3D virtual stimuli (Wehrle et al., 2000; Ambadar et al., 2005; Bould and Morris, 2008). Moreover, recognizing positive expressions is easier than recognizing negative ones for both human and robot performed facial expressions under different conditions. The subjects’ psychophysiological measures were analyzed as indicators of the general autonomic nervous system activity (Lanatà et al., 2011), which can be induced by challenging mental tasks (Stein et al., 1994; Sztajzel, 2004; Dawson et al., 2007). The results demonstrated that interpreting facial expressions of a humanoid robot does not alter the subjects’ psychophysiological states in a different way than interpreting those of human 2D photos and 3D models.

In addition to the exclusion of the expression of happiness, two factors may have influenced the statistical analysis: the small size of the sample and the extended forced-choice paradigm. This work represents a preliminary study of the expression recognition of our robot and its results are encouraging for future experiments.

Future works will aim at improving the performance of the robot by creating dynamic facial expressions and associating them with basic vocalizations and movement of the head/neck. This study highlighted that generating emotional facial expressions is a challenging task that requires high-fidelity reproduction and a deep expertise in animatronic- and artistic-related disciplines. The new expression dataset will be designed by extending the current one with the contribution of artists specialized in facial portrait and sculpture. The problem encountered with the servo motor will be fixed in order to include the happiness expression in the dataset. Moreover, an improved protocol and questionnaire will be designed.

## Conflict of Interest Statement

The authors declare that the research was conducted in the absence of any commercial or financial relationships that could be construed as a potential conflict of interest.

## Footnotes

^1^ARTNATOMY (http://www.artnatomia.net), Victoria Contreras Flores, Spain, 2005
